# Supplementary material for: Polymorphisms in Human IL4, IL10, and TNF Genes Are Associated with an Increased Risk of Developing NSAID-Exacerbated Respiratory Disease
Source: Genes (Basel). 2022 Mar 28;13(4):605. doi: 10.3390/genes13040605 (PMC9031626; doi:10.3390/genes13040605)
Supplement: Supplementary file 1 [file genes-13-00605-s001.zip › genes-1639123-supplementary.pdf]

Supplementary Materials

Supplementary Table S1: Allele and genotypic frequency distribution in the control population and in the NSAID hypersensitivity group.

Gene	Chance	Allele frequency (C)	Allele frequency (NH)	Genotype frequency (C)	Genotype frequency (NH)
IL1A	-889 C>T	C: 1.00<br>T: 0.00	C: 0.98<br>T: 0.02	CC: 1.00<br>CT: 0.00	CC: 0.7<br>CT: 0.03
IL1B	-511 C>T	C: 0.70<br>T: 0.30	C: 0.69<br>T: 0.31	CC: 0.48<br>CT: 0.44<br>TT: 0.08	CC: 0.48<br>CT: 0.41<br>TT: 0.11
IL1B	3962 C>T	C: 0.83<br>T: 0.17	C: 0.76<br>T: 0.24	CC: 0.70<br>CT: 0.26<br>TT: 0.04	CC: 0.57<br>CT: 0.40<br>TT: 0.03
IL1R1	*pst1 C>T*	C: 0.67<br>T: 0.33	C: 0.73<br>T: 0.27	CC: 0.45<br>CT: 0.44<br>TT: 0.11	CC:0.54<br>CT: 0.39<br>TT: 0.07
IL1RN	*mspa1 T>C*	C: 0.24<br>T: 0.76	C: 0.32<br>T: 0.68	CC: 0.05<br>CT: 0.38<br>TT: 0.57	CC: 0.07<br>CT: 0.50<br>TT: 0.43
IL2	-330 T>G	G: 0.32<br>T: 0.68	G: 0.33<br>T: 0.67	GG: 0.10<br>GT: 0.43<br>TT: 0.47	GG: 0.15<br>GT: 0.36<br>TT: 0.49
IL2	166 G>T	G: 0.72<br>T: 0.28	G: 0.64<br>T: 0.26	GG: 0.51<br>GT: 0.41<br>TT: 0.08	GG: 0.44<br>GT: 0.40<br>TT: 0.16
IL4	-1098 T>G	G: 0.07<br>T: 0.93	G: 0.14<br>T: 0.86	GG: 0.03<br>GT: 0.08<br>TT: 0.89	GG: 0.00<br>GT: 0.27<br>TT: 0.73
IL4	-589 C>T	C: 0.86<br>T: 0.14	C: 0.74<br>T: 0.26	CC: 0.75<br>CT: 0.21<br>TT: 0.04	CC: 0.51<br>CT: 0.47<br>TT: 0.02
IL4	-33 C>T	C: 0.86<br>T: 0.14	C: 0.82<br>T: 0.18	CC: 0.75<br>CT: 0.22<br>TT: 0.03	CC: 0.67<br>CT: 0.29<br>TT: 0.04
IL4R	1092 A>G	A: 0.77<br>G: 0.23	A: 0.81<br>G: 0.19	AA: 0.60<br>AG: 0.33<br>GG: 0.07	AA: 0.64<br>AG: 0.34<br>GG: 0.02
IL6	-174 G>C	C: 0.38<br>G: 0.62	C: 0.30<br>G: 0.70	CC: 0.15<br>CG: 0.46<br>GG: 0.39	CC: 0.06<br>CG: 0.48<br>GG: 0.46
IL6	nt565 G>A	A: 0.37<br>G: 0.63	A: 0.27<br>G: 0.73	AA: 0.14<br>AG: 0.46<br>GG: 0.40	AA: 0.04<br>AG: 0.47<br>GG: 0.49
IL10	-592 C>A	A: 0.31<br>C: 0.69	A: 0.30<br>C: 0.70	AA: 0.12<br>AC: 0.38<br>CC: 0.50	AA: 0.02<br>AC: 0.55<br>CC: 0.43
IL10	-819 C>T	C: 0.67<br>T: 0.33	C: 0.69<br>T: 0.31	CC: 0.49<br>CT: 0.36	CC: 0.43<br>CT: 0.53

				TT: 0.15	TT: 0.04
IL10	-1082 A>G	A: 0.67<br>G: 0.33	A: 0.67<br>G: 0.33	AA: 0.46<br>AG: 0.43<br>GG: 0.11	AA: 0.36<br>AG: 0.60<br>GG: 0.04
IL12B	-1188 A>C	A: 0.83<br>C: 0.17	A: 0.73<br>C: 0.27	AA: 0.71<br>AC: 0.25<br>CC: 0.04	AA: 0.54<br>AC: 0.38<br>CC: 0.08
IFN	874 A>T	A: 0.65<br>T: 0.35	A: 0.54<br>T: 0.46	AA: 0.51<br>AT: 0.27<br>TT: 0.22	AA: 0.32<br>AT: 0.45<br>TT: 0.23
TGFB1	c10 T>C	C: 0.41<br>T: 0.59	C: 0.49<br>T: 0.51	CC: 0.18<br>CT: 0.47<br>TT: 0.35	CC: 0.22<br>CT: 0.54<br>TT: 0.24
TGFB1	c25 G>C	A: 0.25<br>G: 0.75	C: 0.06<br>G: 0.94	CC: 0.04<br>CG: 0.16<br>GG: 0.80	CC: 0.01<br>CG: 0.09<br>GG: 0.90
TNF	-238 G>A	A: 0.07<br>G: 0.93	A: 0.08<br>G: 0.92	AA: 0.01<br>AG: 0.14<br>GG: 0.85	AA: 0.01<br>AG: 0.14<br>GG: 0.85
TNF	-308 G>A	A: 0.06<br>G: 0.94	A: 0.25<br>G: 0.75	AA: 0.01<br>AG: 0.10<br>GG: 0.89	AA: 0.06<br>AG: 0.38<br>GG: 0.56

C: controls; NH: NSAID hypersensitivity

Supplementary Table S2: Statistical analysis of the allelic and genotypic distributions of all the SNPs included in the study in NSAID hypersensitivity patients compared with controls.

SNP	# p-value (Allelic)	# p-value (Genotypic)	SNP	# p-value (Allelic)	# p-value (Genotypic)
**rs2234650**	0.17	0.40	**rs1800471**	**0.043***	0.19
**rs1143634**	0.09	0.11	**rs2243248**	**0.023***	**<0.001***
**rs16944**	0.74	0.76	**rs2243250**	**0.002***	**<0.001***
**rs1800587**	0.34	0.34	**rs2070874**	0.26	0.41
**rs2430561**	0.12	0.09	**rs2069762**	0.84	0.48
**rs3212227**	**0.013***	**0.047***	**rs2069763**	0.10	0.14
**rs1801275**	0.34	0.37	**rs1800795**	0.08	0.13
**rs315952**	0.06	0.11	**rs1800797**	**0.041***	**0.040***
**rs1800629**	**<0.001***	**<0.001***	**rs1800896**	0.88	**0.026***
**rs361525**	0.78	0.91	***rs1800871***	0.59	**0.006***
**rs1982073**	0.09	0.18	**rs1800872**	0.79	**0.008***

Fisher's p-value for comparing the group of NSAID hypersensitivity patients patients with the control group.***p-value < 0.05**

A.

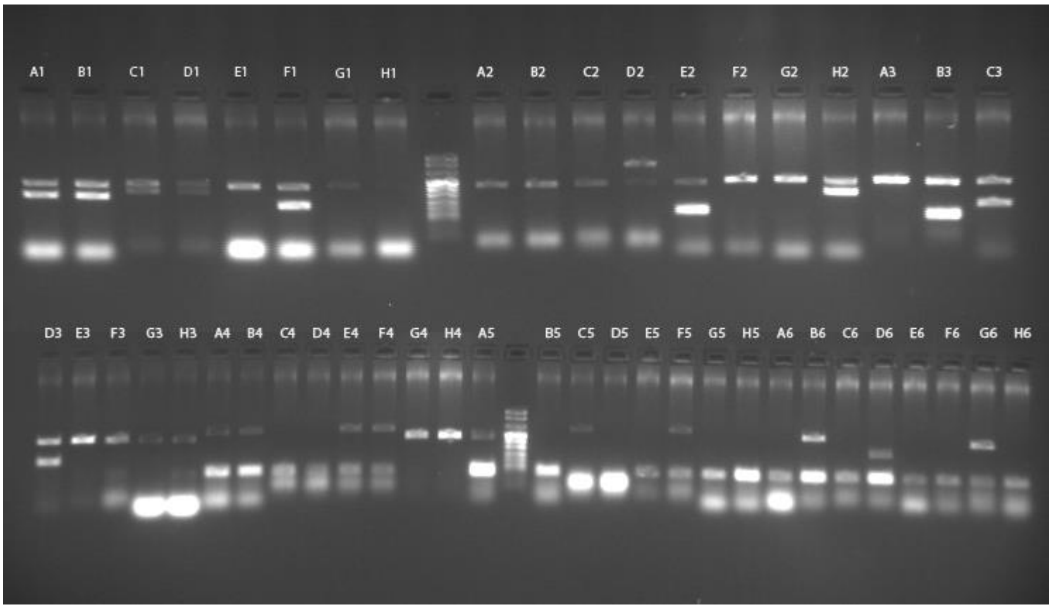

B.

	1	2	3	4	5	6
A	IL-1R<br>T/1970<br>**288**	g-IFN<br>T/874<br>**277**	TNFa<br>A/-308///G/-238<br>**110**	IL4<br>T/-1098//C/-590<br>**557**	IL6<br>C/-174//G565<br>**426**	IL10<br>A/-1082//A/-592<br>**530**
B	IL-1R<br>C/1970<br>**288**	g-IFN<br>A/874<br>**277**	TNFa<br>G/-308///G/-238<br>**110**	IL4<br>T/-1098//T/-590<br>**557**	IL6<br>G/-174//G565<br>**427**	IL10<br>A/-1082//C/-592<br>**530**
C	IL-1b<br>C/+3962<br>**336**	IL-12<br>A/-1188<br>**802**	TGFb1<br>T/10<br>**195**	IL2<br>T/-330//T/166<br>**569**	IL4<br>C/-590//C/-33<br>**610**	IL10<br>A/-1082//T/-819<br>**305**
D	IL-1b<br>T/+3962<br>**336**	IL-12<br>C/-1188<br>**802**	TGFb1<br>C/10<br>**195**	IL2<br>G/-330//T/166<br>**569**	IL4<br>C/-590//T/-33<br>**610**	IL10<br>A/-1082//C/-819<br>**305**
E	IL-1b<br>T/-511<br>**215**	IL-4Ra<br>A/1902<br>**143**	TGFb1<br>T/10-C/25<br>**80**	IL2<br>G/-330//G/166<br>**564**	IL4<br>T/-590//C/-33<br>**610**	IL10<br>G/-1082//C/-592<br>**530**
F	IL-1b<br>C/-511<br>**215**	IL-4Ra<br>G/1902<br>**143**	TGFb1<br>T/10-G/25<br>**80**	IL2<br>T/-330//G/166<br>**562**	IL4<br>T/-590//T/-33<br>**610**	IL10<br>G/-1082//C/-819<br>**305**
G	IL-1a<br>C/-889<br>**220**	IL-1RA<br>C/11100<br>**297**	TGFb1<br>C/10-C/25<br>**80**	TNFa<br>A/-308//A/-238<br>**110**	IL4<br>G/-1098//C/-590<br>**557**	IL6<br>C/-174//A/565<br>**428**
H	IL-1a<br>T/-889<br>**220**	IL-1RA<br>T/11100<br>**297**	TGFb1<br>C/10-G/25<br>**80**	TNFa<br>G/-308//A/-238<br>**110**	IL4<br>G/-1098//T/-590<br>**557**	IL6<br>G/-174//A565<br>**428**

(gray)	Control 440pb
(white)	Control 89

Supplementary Figure S1. Results of PCR-SSP genotyping analysis. A: Visualization with UV light of the 22 polymorphisms through bands in an electrophoresis gel corresponding to a patient. **B:** Results interpretation template. Each box corresponds to each of the wells of image A. In each box the amplifying allele is indicated, as well as the size in base pairs of the product band. In the gray shaded boxes the size of the control band is 440 bp, and in the white boxes the control band is 89 bp.
